# PS-YOLO-seg: A Lightweight Instance Segmentation Method for Lithium Mineral Microscopic Images Based on Improved YOLOv12-seg

**DOI:** 10.3390/jimaging11070230

**Published:** 2025-07-10

**Authors:** Zeyang Qiu, Xueyu Huang, Zhicheng Deng, Xiangyu Xu, Zhenzhong Qiu

**Affiliations:** 1 Yichun Lithium New Energy Industry Research Institute, Jiangxi University of Science and Technology, Yichun 336000, China; qq2462218642@163.com; 2 School of Software Engineering, Jiangxi University of Science and Technology, Nanchang 330013, China; dengzhicheng@jxust.edu.cn (Z.D.); xuxiangyu@jxust.edu.cn (X.X.); 3 School of Electronic Information Industry, Jiangxi University of Science and Technology, Ganzhou 341600, China; 4 Jiangxi Yongxing Special Steel New Energy Technology Co., Ltd., Yichun 336300, China; yxxny@yongxingbxg.com

**Keywords:** instance segmentation, lepidolite, YOLOv12-seg, lightweight network, edge deployment, microscopic imaging

## Abstract

Microscopic image automatic recognition is a core technology for mineral composition analysis and plays a crucial role in advancing the intelligent development of smart mining systems. To overcome the limitations of traditional lithium ore analysis and meet the challenges of deployment on edge devices, we propose PS-YOLO-seg, a lightweight segmentation model specifically designed for lithium mineral analysis under visible light microscopy. The network is compressed by adjusting the width factor to reduce global channel redundancy. A PSConv-based downsampling strategy enhances the network’s ability to capture dim mineral textures under microscopic conditions. In addition, the improved C3k2-PS module strengthens feature extraction, while the streamlined Segment-Efficient head minimizes redundant computation, further reducing the overall model complexity. PS-YOLO-seg achieves a slightly improved segmentation performance compared to the baseline YOLOv12n model on a self-constructed lithium ore microscopic dataset, while reducing FLOPs by 20%, parameter count by 33%, and model size by 32%. Additionally, it achieves a faster inference speed, highlighting its potential for practical deployment. This work demonstrates how architectural optimization and targeted enhancements can significantly improve instance segmentation performance while maintaining speed and compactness, offering strong potential for real-time deployment in industrial settings and edge computing scenarios.

## 1. Introduction

Lepidolite is a key raw material for lithium extraction from hard rock ores. As an important strategic mineral resource, lithium-ion batteries made from lepidolite are widely used in core fields such as electric vehicles, energy storage systems, and smart devices, playing an irreplaceable role in the ongoing global energy transition and low-carbon development [1,2,3,4]. In recent years, driven by the global trends of electrification and digitalization, the demand for lithium resources has continued to rise. According to the International Energy Agency (IEA), global lithium consumption is expected to increase from the current amount of less than 700,000 tons of lithium carbonate equivalent (LCE) to over 2 million tons by 2030 [5], resulting in a several-fold expansion in demand in the short term. This structural contradiction of “high demand–low security” highlights the importance of efficient exploration, mining, and precise identification of lithium mineral resources [6,7]. Against this backdrop, enhancing the intelligent recognition and precise sorting capabilities of lithium ores is not only a critical pathway to improving resource utilization and beneficiation efficiency, but also has significant implications for ensuring the security of national strategic mineral resources and promoting the sustainable development of the new energy industry [8,9].

In recent years, computer vision technology has made significant progress in the field of mineral identification and classification, gradually addressing the shortcomings of traditional manual methods, such as destructive sampling, lengthy detection cycles, and high operational and maintenance costs [10,11]. However, traditional computer vision techniques—typically relying on hand-crafted features such as color, texture, and edge descriptors—often struggle with the complex and highly variable visual characteristics of mineral microscopic images. These methods lack robustness against uneven lighting, weak boundaries between mineral phases, and overlapping regions, which are common in thin section images [12]. Moreover, their generalization ability is limited, and they require extensive domain expertise for feature engineering, making them less suitable for automated, large-scale mineral analysis [13]. For example, Feng et al. [14] proposed a handcrafted feature extraction pipeline for flotation froth image analysis, which involved multiple preprocessing steps, such as image enhancement, segmentation using improved watershed algorithms, and optical flow tracking. While effective under specific conditions, such approaches are labor-intensive, sensitive to parameter settings, and offer limited adaptability to complex scenarios. These challenges highlight the need for deep learning-based approaches that are capable of learning hierarchical representations directly from raw image data in an end-to-end manner.

Compared to traditional computer vision methods, deep learning has shown superior performance in mineral analysis by automatically learning hierarchical features from raw images. These models are more robust to lighting variations, weak boundaries, and overlapping regions, and they reduce the need for manual feature engineering [15]. Despite these advancements, there are still several challenges in applying such technologies to lithium ore detection. On one hand, lithium ores often exhibit complex and variable surface textures under the microscope, and slight morphological differences between mineral blocks further complicate quality identification [16]. On the other hand, to enhance the model’s discriminative power, existing deep learning methods often rely on deeper network architectures, leading to a dramatic increase in model parameters. This not only significantly increases the system’s computational load, but also raises higher requirements for the real-time performance in industrial scenarios, limiting the deployment efficiency and practicality of such models on edge devices [17,18,19]. As highlighted in a recent systematic review by Liu et al. [20], deploying deep learning models on embedded platforms remains a major challenge due to limited computational and memory resources. Their study summarized over a decade of research and emphasized the growing need for lightweight architectures and efficiency-driven optimization techniques to support low-latency, high-performance applications in resource-constrained environments. Therefore, how to balance lightweight design and real-time processing while maintaining high recognition accuracy is equally crucial in the current field of lithium ore recognition technology.

Recently, the You Only Look Once (YOLO) series has emerged as one of the most widely used architectures for real-time object detection and instance segmentation due to its end-to-end design and favorable trade-off between speed and accuracy [21]. YOLO models typically employ stacked convolutional layers to extract hierarchical features from input images. However, as pointed out earlier, as the network depth increases to improve representational power, issues such as redundant parameters, high computational cost, and feature degradation during downsampling have become more pronounced. These limitations hinder the effective deployment of YOLO-based models on edge devices, especially in fine-grained tasks like lithium mineral segmentation under microscopic conditions.

To overcome the limitations of traditional methods in terms of accuracy and efficiency, and to enhance both the deployment efficiency of existing models and their adaptability in microscopic environments, this paper proposes an improved YOLOv12-seg network architecture for efficient instance segmentation of lithium mineral components. The model has undergone streamlined optimization in its overall architecture design, introducing dedicated downsampling operations and integrating an enhanced feature extraction module. This design improves the accuracy and speed of ore category recognition and boundary contour extraction, ensuring the segmentation performance of the model while effectively managing computational resources and optimizing inference efficiency. The model is particularly suitable for deployment in resource-constrained environments, and is ideal for real-time applications on edge computing devices, enabling rapid intelligent analysis of ore quality.

The main contributions and methods of this study are as follows:

1. Through a systematic preparation process, including industrial site sampling, microscope imaging, and data optimization preprocessing, we independently constructed a lithium mineral microscopic image dataset containing tens of thousands of instances from the following three categories: feldspar, quartz, and lepidolite.
2. By adjusting the width factor of the network, we proportionally compressed the global channel count, constructing a more compact YOLOv12t-seg network compared to the original model. This effectively eliminated channel redundancy in small-scale tasks, significantly reducing the number of parameters while maintaining the core performance of the model in segmentation tasks.
3. We introduced PSConv to replace conventional convolutions, significantly increasing the receptive field in the downsampling operation while further reducing the number of parameters. Additionally, we incorporated PSConv into C3k2, creating a new feature extraction module; C3k2-PS, to enhance spatial distribution perception; and feature extraction capability for faint targets in the microscopic environment.
4. By streamlining the original segmentation head structure and eliminating redundant depthwise separable convolution operations, we proposed an efficient Segment-Efficient segmentation head. This approach effectively reduces model complexity while enhancing deployment efficiency on resource-constrained devices.

The rest of this paper is structured as follows: Section 2 reviews the relevant literature on lithium mineral quality analysis and identifies existing challenges. Section 3 introduces the proposed improved model and details the specific modifications. Section 4 describes the dataset preparation, experimental setup, parameter settings, and the quantitative evaluation metrics. Section 5 analyzes the experimental results, presenting data and conclusions from various comparative experiments. Finally, Section 6 summarizes the findings and contributions of this study, and discusses the next steps, as well as potential directions for future research.

## 2. Related Work

Traditional lithium mineral grade analysis methods mainly rely on physicochemical spectral analysis and chemical quantitative techniques. For example, Fabre et al. [22] identified distinct absorption features of lepidolite in the ultraviolet near-infrared spectra using reflectance spectroscopy, analyzing its quality and distinguishing it from other muscovite-type minerals. Fu et al. [23] developed a chemical phase analysis method for lithium in pegmatitic lithium ores by selectively leaching iron–lithium mica, lepidolite, and spodumene using dilute hydrochloric acid, concentrated sulfuric acid, and hydrofluoric acid. Wang et al. [24] used a portable field spectrometer to test and analyze the spectral reflectance of typical lithium-bearing rocks and mineral samples, revealing the characteristics of pegmatitic lithium ores from different regions. Although these methods provide more accurate lithium mineral analysis results, they typically rely on manual execution of multiple steps, leading to complexities in procedures, low time efficiency, and high labor intensity. These limitations significantly restrict their application in large-scale industrial deployment and real-time monitoring.

With the advancement of image analysis techniques, computer vision and machine learning methods have emerged as promising tools for mineralogical research [25]. Early studies primarily adopted traditional computer vision approaches that relied on handcrafted feature extraction—such as Local Binary Patterns (LBP), Histogram of Oriented Gradients (HOG), and Scale-Invariant Feature Transform (SIFT)—combined with morphological operations, watershed algorithms, and threshold-based segmentation for mineral grain boundary detection. For example, Han et al. [26] developed a semi-supervised segmentation framework using the GL-SLIC algorithm, which integrates Gabor filters and LBP to perform a superpixel-based analysis on sandstone thin section images, highlighting the potential of classical feature-driven pipelines in petrographic tasks. In addition, machine learning methods combining image or spectral features—such as XGBoost and clustering algorithms—have been employed to classify mineral types. For instance, Ji et al. [27] used the XGBoost machine learning algorithm combined with chemical composition to differentiate various types of serpentine, and analyzed the relationship between the chemical composition of serpentine minerals and their formation environments through the k-means clustering algorithm. Dai et al. [28] proposed an integrated machine learning algorithm that combined laser-induced breakdown spectroscopy (LIBS) and remote sensing spectra for the classification of six types of minerals, demonstrating that the fusion of machine learning can rapidly and reliably perform mineral classification. Despite their successes, both traditional CV and classical machine learning approaches heavily rely on handcrafted features, which require task-specific adjustments for different mineralogical applications. Moreover, these methods are often sensitive to environmental variations and lack the adaptability and robustness offered by recent deep learning techniques when applied under varying conditions.

In contrast, the combination of deep learning algorithms with mineral images overcomes some of the limitations of traditional methods, such as tedious instrument operations and manual modeling, by automatically mining the complex relationship between optical features and mineral grades. Furthermore, they enable non-contact visual inspection for real-time, second-level analysis, providing more efficient solutions for industrial continuous monitoring [29]. For instance, Latif et al. [30] proposed a method that combined superpixel segmentation with a deep residual network (ResNet) to identify rock-forming minerals in optical microscopy images. Their model achieved a high validation accuracy of 90.5%, demonstrating the superior ability of deep architectures to automatically learn complex textural patterns that traditional handcrafted features often fail to capture. Similarly, Song et al. [31] refined the encoder–decoder structure of U-Net for electron microscopy (EM) images, achieving a Rand index of 0.916. This result highlights the strength of deep learning in delineating fine-grained mineral boundaries, which are typically challenging for conventional image processing or shallow learning algorithms. These studies exemplify how deep learning surpasses traditional approaches by enabling end-to-end feature learning, greater robustness to imaging variability, and improved segmentation accuracy in mineralogical contexts.

Despite their merits, most models are resource-heavy and modality-specific, hindering edge deployment. Recognizing these limitations, recent studies have increasingly emphasized the importance of lightweight models that can operate efficiently on edge devices without compromising accuracy. For instance, Li et al. [32] reported that full-scale YOLOv5 models [33] exhibited excessive latency and energy consumption in real-world deployment, whereas their streamlined variants significantly reduced the computational burden. Similarly, Wang et al. [34] demonstrated that, compared to baseline models such as YOLOv4-tiny [35] and MobileNet SSD [36], simplified architectures improved inference speed and were better-suited for mobile applications. These findings underscore the critical role of model complexity in practical deployment efficiency, particularly in industrial and field-based scenarios. Additionally, there are limited methods using deep learning instance segmentation for lithium mineral microscopic image analysis, indicating a need for further advancement in this area. Therefore, the aim of this study is to propose a segmentation model for lithium mineral microscopic images that combines high accuracy with lightweight features, enabling both high segmentation performance and efficient deployment on edge devices to enhance practical industrial applications.

## 3. Methods

### 3.1. The Original YOLOv12-seg Network

YOLOv12 [37] is an advanced real-time object detection framework centered on an attention mechanism, designed to overcome the limitations of traditional YOLO series architectures, which primarily rely on convolutional structures. It enhances the overall performance by balancing precision and speed.

**Figure 1:**
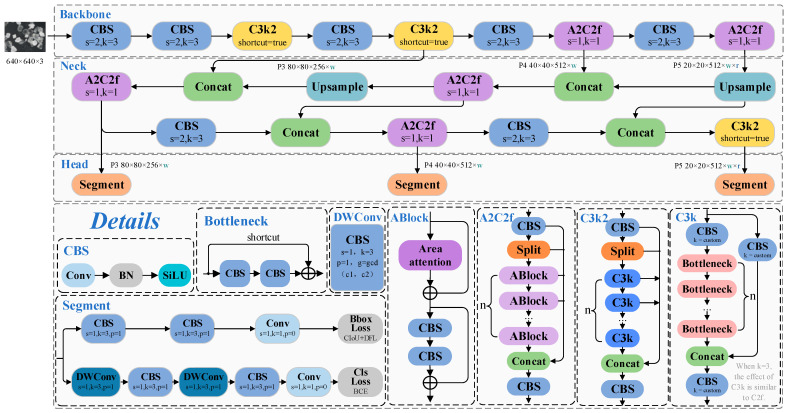
Schematic diagram of the YOLOv12-seg network architecture. The variables w, d, and r represent the width factor, depth factor, and ratio, respectively.

The improvements in YOLOv12 compared to previous versions mainly include the following: (i) The introduction of the Area Attention module, which constructs an enhanced feature extraction module, A2C2f. This module effectively reduces computational complexity by dividing regions horizontally or vertically, and incorporates Flash Attention technology to optimize memory access efficiency, thus improving inference speed. (ii) The proposal of the Residual Efficient Layer Aggregation (R-ELAN) structure, which strengthens feature fusion capabilities and enhances training stability, addressing the gradient blockage issues in the original ELAN module [38]. (iii) The adjustment of the MLP ratio and the introduction of large-kernel separable convolutions to replace the positional encoding module, reducing redundant computations while maintaining position awareness. Although YOLOv12 demonstrates a superior performance due to its advanced architecture, it still faces challenges related to model complexity and deployment difficulties, leaving room for further optimization and compression. The model’s architecture and relevant details are shown in Figure 1.

### 3.2. The Improved PS-YOLO-seg Network

Considering the precision requirements and computational constraints associated with lithium mineral segmentation tasks in microscopic environments, this study proposes a high-accuracy and lightweight segmentation network based on the YOLOv12-seg baseline. The proposed model retains the fundamental components of YOLOv12-seg, including the backbone, neck, and head, while incorporating several targeted optimizations.

**Figure 2:**
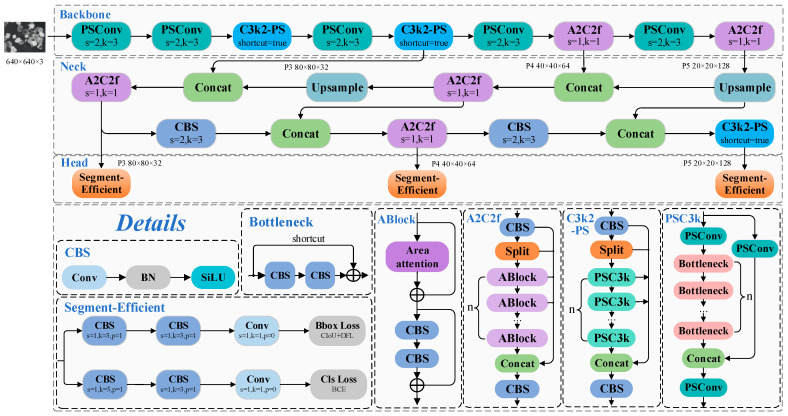
Schematic diagram of the PS-YOLO-seg network architecture.

First, the width factor is adjusted to compress the global number of channels to half of the original configuration, significantly reducing both the parameter count and computational overhead. Second, a convolutional variant called PSConv (Pinwheel-shaped Convolution) [39] is introduced into the backbone to replace standard convolutional layers. PSConv enhances the computational efficiency by decoupling spatial and channel-wise filtering into parallel branches, effectively expanding the receptive field without increasing parameter burden. To improve fine-grained feature extraction under the microscopic context, we further enhance the C3k2 module—a residual-based feature extraction block—by integrating PSConv into its internal layers. The resulting module, named C3k2-PS, boosts the spatial representation capability while maintaining a compact structure, making it better-suited for distinguishing subtle textural variations in lithium mineral imagery. Additionally, considering the segmentation characteristics of mineral boundaries and the constraints of edge deployment, we redesign the segmentation head by removing redundant depthwise separable convolutions. The optimized head detection branch, termed Segment-Efficient, adopts a streamlined layout that reduces model complexity while preserving the segmentation accuracy. This refinement improves the inference speed and enhances practical deployability on resource-constrained devices. The overall architecture and selected structural details are illustrated in Figure 2.

The following subsections will provide detailed explanations of the design principles and implementation details of these optimized modules.

### 3.3. YOLOv12t-seg

The YOLOv12 series supports multiple network configurations (e.g., n, s, m, l, x) through adjustable width and depth scaling factors (widen factor and deepen factor), enabling structural flexibility based on task requirements. Specifically, the widen factor regulates the number of channels per layer to control the network width, while the deepen factor increases the repetition of network modules to enhance feature extraction. Although deeper architectures facilitate the learning of complex features, they also substantially increase computational costs.

Given that this study targets the segmentation of lithium-bearing minerals in microscopic images with a limited number of categories, even the smallest model in the YOLOv12 segmentation series (YOLOv12n-seg) exhibits channel redundancy. To further improve model efficiency, this work proposes YOLOv12t-seg, which retains the original network architecture but reduces both the width factor and maximum channel count by half, thereby significantly lowering the number of parameters. Specifically, the number of channels in the Backbone (layers 0 to 8) and Neck (layers 11, 14, 17, and 20) is halved, resulting in a more compact structure compared to baseline models. Table 1 presents a comparison of YOLOv12t-seg with YOLOv12n-seg (baseline) and YOLOv12s-seg in terms of scaling factors and parameter sizes.

### 3.4. PSConv

**Figure 3:**
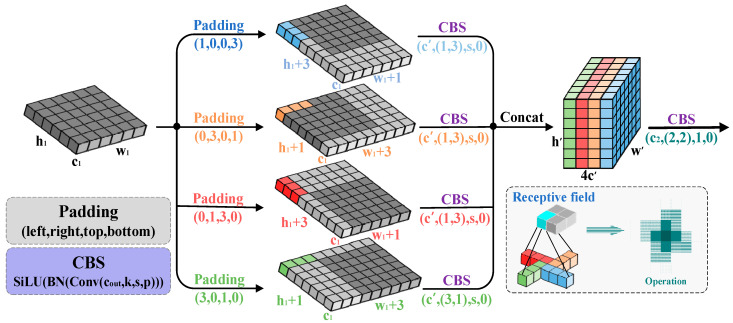
Schematic diagram of PSConv.

To better account for the spatial distribution characteristics of dim, small target pixels in microscopic environments, and to optimize the model’s detection capabilities in such specialized scenarios, a novel lightweight pinwheel-shaped convolution (PSConv) has been introduced. Unlike traditional convolution operations, PSConv uses a pinwheel-shaped kernel structure, which enables more precise capture of the spatial distribution patterns and local features of dim small targets. By incorporating PSConv into the lower layers of the backbone network, the aim is to effectively enhance the response to small targets while maintaining low computational complexity. The operational schematic of PSConv is shown in Figure 3.

Specifically, let the input feature map be denoted as $x\in R^{c_{1}\times h_{1}\times w_{1}}$, where $c_{1}$, $h_{1}$, and $w_{1}$ represent the channel number, height, and width, respectively. To enhance the training stability and nonlinearity of the model, batch normalization (BN) and the sigmoid activation function SiLU are applied after each convolutional operation, forming the CBS structure (Conv-BN-SiLU). The proposed PSConv performs directional convolutions in four distinct orientations. The calculation process is expressed as follows:

(1) $$x_{i}^{\left(c^{\prime },h^{\prime },w^{\prime }\right)}=SiLU(BN(x_{{Padding}_{\left(left,right,top,bottom\right)}}^{\left(c_{1},h_{1},w_{1}\right)}⊗W_{i}^{\left(c^{\prime },k,d\right)}))$$

where “⊗” denotes the convolution operation; i=1,2,3,4, respectively, represent four different directions; and $W_{i}^{\left(c^{\prime },k,d\right)}$ represents convolutional kernels with the size of k×d. The padding parameters are adjusted according to four spatial orientations. The input feature map is partitioned into four branches, and each branch undergoes convolution with directional kernels. The spatial dimensions of the output features are determined by $h^{\prime }={1+h}_{1}/s$, $w^{\prime }={1+w}_{1}/s$, and $c^{\prime }=c_{2}/4$, where $c_{2}$ is the final output channel number of PSConv, and s denotes the stride. The results of the four directional convolutions are concatenated along the channel axis, as follows:

(2) $$x_{inter}^{\left(4c^{\prime },h^{\prime },w^{\prime }\right)}=Concat(x_{1}^{\left(c^{\prime },h^{\prime },w^{\prime }\right)},\ldots ,x_{4}^{\left(c^{\prime },h^{\prime },w^{\prime }\right)}))$$

Subsequently, the concatenated feature map is passed through a 2 × 2 convolutional kernel without padding to refine the features. This step enhances the spatial correlation and resolution of the features by aggregating local responses. The output feature map Y, with the dimensions of $h_{2}=h_{1}/s$, $w_{2}=w_{1}/s$, and $c_{2}$, is computed as follows:

(3) $$Y^{\left(c_{2},h_{2},w_{2}\right)}=SiLU(BN(x_{inter}^{\left(4c_{1},h_{1},w_{1}\right)}⊗W_{i}^{\left(c_{2},2,2\right)}))$$

To highlight the parameter efficiency and spatial modeling capability of PSConv compared to conventional convolutional layers, a quantitative analysis is provided. Assuming the input and output channels are equal, $c_{1}=c_{2}$, and the kernel size is k=3, the number of parameters required by standard convolution and PSConv is approximately

(4) $${Conv}_{params}=c_{1}\times c_{2}\times k^{2}=9c_{1}^{2}$$

(5) $${PSConv}_{params}=4\times (c_{1}\times (\frac{c_{2}}{4})\times 3\times 1)+4c_{1}c_{2}=7c_{1}^{2}$$

The computational results indicate that PSConv expands the receptive field to 177% of that of standard convolution while using less than 80% of the parameters. PSConv utilizes asymmetric padding to construct quasi-symmetric non-block convolutions along the horizontal and vertical directions, effectively expanding the receptive field area while maintaining a relatively low number of model parameters. This enhances the downsampling efficiency in the lower layers, thereby ensuring a balanced trade-off between computational efficiency and model performance.

### 3.5. C3k2-PS

**Figure 4:**
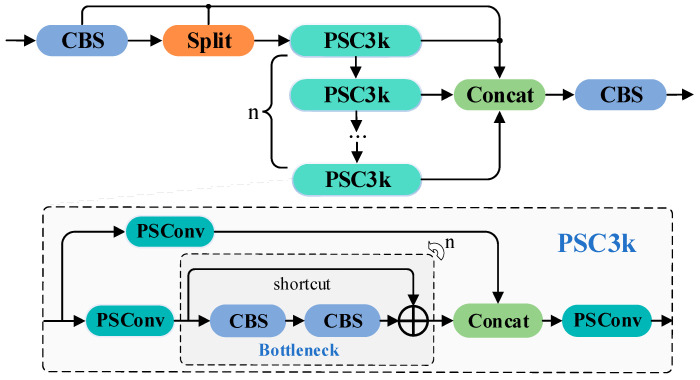
Structural schematic of the C3k2-PS and PSC3k.

Compared to the previous feature extraction modules, C3 and C2f, the C3k2 offers a more flexible feature flow path, enhancing the feature representation capability. However, for specific tasks, such as fine segmentation of lithium minerals in microscopic environments, there remains room for optimization. To further improve the ability to capture texture details in lithium mineral microscopic images, we integrated PSConv into the C3k bottleneck module of YOLOv12, forming a novel PSC3k bottleneck module, and further proposed an improved feature extraction module, C3k2-PS. This module specifically targets the extraction of faint mineral textures in microscopic environments. By utilizing the efficient pinwheel-shaped convolution structure of PSConv, it optimizes the receptive field and feature extraction performance, thereby enhancing segmentation capability for lithium minerals. The structural details are shown in Figure 4.

The C3k2-PS module builds upon the C3k2 module’s branching structure, further enhancing the diversity and robustness of feature extraction in specialized scenarios. In this design, input features pass through an initial convolutional layer (CBS) and are then split into multiple subspaces. One subspace is directly forwarded to the final concatenation layer, while the other enters multiple PSC3k bottleneck modules. In each PSC3k bottleneck, features undergo PSConv convolution, which improves the extraction of small object features through a large receptive field and central point weights. After this, features pass through several Bottleneck structures for further transformation and are merged with the output of another PSConv branch via Concat. A final PSConv layer refines the feature representation. This design allows the C3k2-PS module to effectively capture faint mineral textures in microscopic images. The final step aggregates the features processed by the PSC3k bottlenecks to produce the output feature map. Compared to the traditional C3k2, the C3k2-PS, with its pinwheel-shaped parallel padding convolutions, is more efficient in small object detection, particularly in environments with small and faint targets, improving segmentation accuracy and computational efficiency. This design is especially suitable for resource-constrained environments, such as embedded devices, ensuring a balance between real-time performance and accuracy.

### 3.6. Segment-Efficient

**Figure 5:**
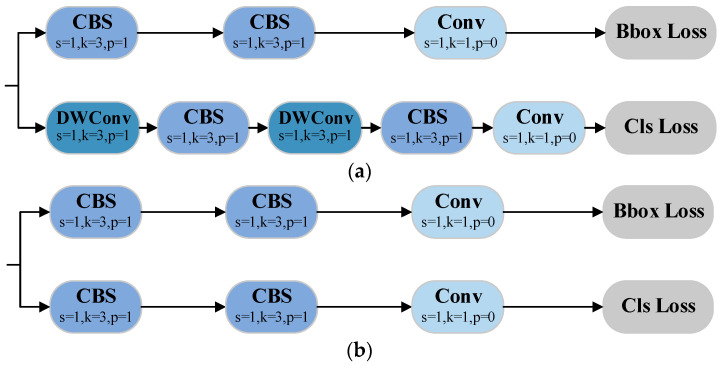
Comparison of segmentation head structures. (**a**) Segmentation head of YOLOv12-seg; (**b**) Segment-Efficient of PS-YOLO-seg.

Unlike segmentation tasks involving complex backgrounds and multiple classes, this study focuses on the segmentation of a small number of lithium mineral categories in a single microscopic environment. Under high-magnification microscopy, the boundaries between mineral blocks and the background can typically be observed clearly and effectively. Therefore, the original segmentation head contains redundant operations when generating bounding boxes and class labels after feature extraction. To better suit the specific needs of this scenario, we redesigned the segmentation head and proposed the Segment-Efficient module. This module optimizes the redundant operations in the original segmentation head by removing depthwise separable convolutions (DWConv) [40]. The revised head structure is compared to the original in Figure 5.

The Segment-Efficient module maintains the original decoupling approach between detection boxes and classification tasks, enabling independent execution of localization, classification, and semantic segmentation. This allows the network to learn distinct feature representations for each task, avoiding feature competition and improving overall accuracy. Moreover, by eliminating unnecessary convolution operations, the head process is optimized, accelerating model inference and reducing computational resource consumption, thus enhancing the feasibility of efficient deployment in resource-constrained environments.

**Figure 6:**
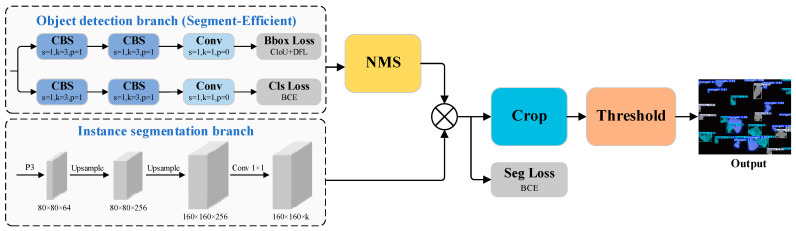
Structural Diagram of the Head section with Segment-Efficient Integration.

Inspired by the modular design of YOLACT [41], the proposed segmentation head employs a dual-branch head architecture to simultaneously perform object detection and instance segmentation. Specifically, the detection branch is responsible for predicting category scores, bounding box offsets, and a set of mask coefficients, which contribute to the computation of classification and localization losses. In parallel, the segmentation branch produces a set of shared prototype masks at a global level. After applying non-maximum suppression (NMS) to filter overlapping detections, the final instance-specific masks are derived by linearly combining the prototype masks with the corresponding mask coefficients through matrix multiplication. This is followed by binary cross-entropy (BCE) loss optimization. The resultant masks are refined using cropping and thresholding operations to generate the final segmentation outputs. A detailed illustration of the segmentation head incorporating the Segment-Efficient module is provided in Figure 6.

## 4. Data and Experimental Preparation

### 4.1. Dataset Construction

**Figure 7:**
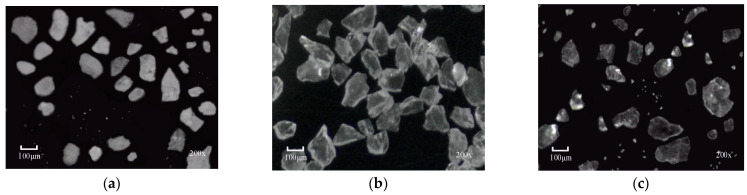
Lithium mineral micrographs. (**a**) Feldspar; (**b**) quartz; (**c**) lepidolite.

The lithium mineral samples used in this study were sourced from the beneficiation plant of Jiangxi Yongxing Special Steel New Energy Technology Co., Ltd. (Yichun, China). These samples consist of high-quality mineral powders that have been ground and dried on the industrial production line, containing the following three main components: feldspar, quartz, and lepidolite. Imaging was performed using a KO series microscope (model: KOMD0745; LED illumination: 10W; Shenzhen, China) with the following configuration: 3840 × 2160 pixel resolution, 120 mm working distance, and a 0.5× objective lens (focal length: 0.7; magnification: 200×). Using the fine imaging technology of this device, multiple high-resolution microscopic images were captured, providing detailed information on the mineral block’s various forms, textures, and edge features. Figure 7 shows a selection of the captured lithium mineral microscopic images.

**Figure 8:**
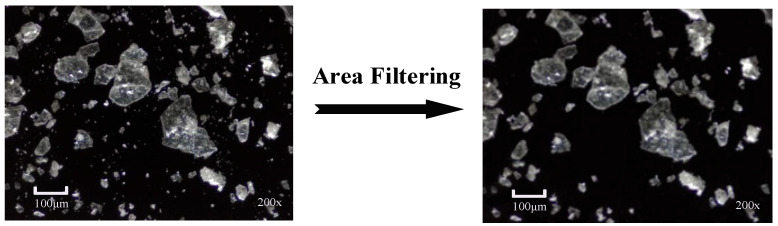
Schematic of micrographs optimization preprocessing.

To remove excessively small fragments and focus on the primary mineral block targets, this study applied a unified data optimization process to the existing lithium mineral microscopic images. Since these small fragments are typically noise or non-representative debris without significant geological or industrial value, retaining them may not only introduce labeling ambiguity, but also unnecessarily increase the computational overhead. First, a small region area filtering algorithm [42] was used to analyze and statistically count the number of connected pixels in the image. Then, based on the resolution of the actual application, the minimum effective region size (filtering threshold) was set to 100 pixels to filter out tiny fragments. Finally, Gaussian filtering was applied to repair any edge artifacts or holes that may have been introduced during the filtering process. Figure 8 presents an example of this data preprocessing, visually showing the comparison before and after optimization.

After data preprocessing, the Labelme image annotation tool [43] was used for manual labeling. During the labeling process, irregular polygons were drawn along the edges of the mineral block contours, and each mineral block was assigned the corresponding category label. The annotation results were saved in the JSON format and then uniformly converted into the TXT format required by the YOLO model. Using this method, a lithium mineral microscopic image dataset was created, containing 15,682 mineral block instances and their corresponding annotations, including 4826 feldspar blocks, 6080 quartz blocks, and 4776 lepidolite blocks. Finally, the dataset was randomly split into training, validation, and test sets in a 7:2:1 ratio. The specific distribution is shown in Table 2.

### 4.2. Experimental Environment

The experiments in this study were conducted on a standardized experimental platform, with the following hardware specifications: the central processing unit (CPU) is a 12th generation Intel Core i5-12490F (3.00 GHz, Intel Corporation, Ho Chi Minh City, Vietnam), and the graphics processing unit (GPU) is an NVIDIA GeForce RTX 4060 (8GB VRAM, NVIDIA Corporation, Santa Clara, CA, USA). The software environment includes Windows 11 Professional Edition (System version: 23H2) as the operating system, with the programming environment based on Python 3.8.19. Deep learning experiments utilized the PyTorch 1.12 framework, with GPU acceleration enabled via CUDA 11.3.

All experiments used a unified training configuration, as follows: a total of 150 training epochs, with a batch size of 8. The optimizer employed was stochastic gradient descent (SGD), with a momentum coefficient of 0.937, an initial learning rate of 0.01, and a weight decay factor of ${5\times 10}^{-4}$. An early stopping mechanism was used during training, with a patience threshold set to 50 epochs. These settings were mainly based on commonly used practices in similar-scale and domain-related detection tasks. During most of the training process, the Mosaic data augmentation technique was applied to enhance data diversity and improve model generalization. To enhance the stability of model convergence, the Mosaic data augmentation strategy was disabled during the final 10 epochs.

### 4.3. Evaluation Criteria

To systematically evaluate the model’s segmentation performance and lightweight characteristics based on practical application requirements, eight core metrics were used for a comprehensive assessment. The model’s accuracy is represented by Precision (P) and mean Average Precision at an IoU threshold of 50 (mAP50). Precision (P) quantifies the model’s ability to predict positive samples, representing the proportion of true-positive samples among all predicted positive samples. mAP50 is the mean average precision at an IoU threshold of 0.50 for bounding box or pixel segmentation, providing an overall performance evaluation across all categories. The mathematical formulas for these metrics are as follows:

(6) $$P=\frac{TP}{TP+FP}\times 100\%$$

(7) $$mAP50=\frac{1}{N}\sum_{i=1}^{N}\int_{0}^{1}P_{i}(r_{i})dr_{i}\times 100\%$$

In the formulas, TP represents the number of correctly predicted positive samples and FP represents the number of incorrectly predicted positive samples, where $P_{i}(r_{i})$ denotes the interpolated precision value at a recall rate of $r_{i}$ for the i-th class, and N represents the total number of classes. The above metrics are further subdivided into Detection Precision ($P_{box}$), Segmentation Precision ($P_{mask}$), mean Average Precision for bounding boxes (${mAP50}_{box}$), and mean Average Precision for segmentation masks (${mAP50}_{mask}$). These metrics specifically quantify the accuracy of bounding box localization and the accuracy of generated segmentation masks.

In terms of lightweight characteristics, the evaluation is comprehensively carried out through the following four metrics: Floating Point Operations (FLOPs), Frames Per Second (FPS), number of network parameters (Params), and model storage size (Size). Among these, FLOPs and FPS quantify the model’s computational complexity and real-time performance, while Params and Size reflect the model’s spatial complexity and deployment feasibility.

## 5. Experiments and Discussion

### 5.1. Model Training

**Figure 9:**
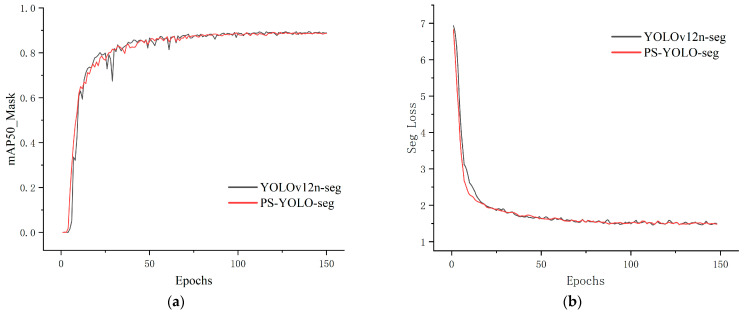
Training curves of YOLOv12n-seg and PS-YOLO-seg. (**a**) ${mAP50}_{mask}$-epochs curve; (**b**) segmentation loss-epochs curve.

The PS-YOLO-seg model was trained on the self-constructed lithium mineral microscopic image dataset using the preset parameter configuration, with YOLOv12n-seg as the baseline model for comparison. The average segmentation accuracy and loss during the training process are shown in Figure 9. In the early stages of training, both models quickly adapted to the dataset, with ${mAP50}_{mask}$ rapidly increasing and the segmentation loss quickly decreasing. Compared to the baseline model, the improved model exhibited a faster and more stable upward trend in performance. In the later stages of training, both models’ metrics began to converge, but the baseline model still showed slight fluctuations, whereas the improved model remained more stable. This indicates that the PS-YOLO-seg has stronger feature extraction and data adaptation capabilities, and the improved module effectively enhanced the model’s ability to segment different types of lithium minerals in microscopic environments.

### 5.2. Ablation Experiments

To systematically verify the effectiveness of each architectural modification, the YOLOv12t-seg model (the channel-scaled version) was used as the base, with the proposed improvements integrated progressively through a staged protocol. Following the principle of controlled variables, all experiments were conducted with the same hyperparameter settings, and several ablation experiments were designed based on mathematical combinations. The specific improvements included the following:

A: Replacing the downsampling operation in the backbone network with PSConv;

B: Replacing the global C3k2 module with the C3k2-PS module;

C: Adopting the lightweight Segment-Efficient segmentation head.

**Figure 10:**
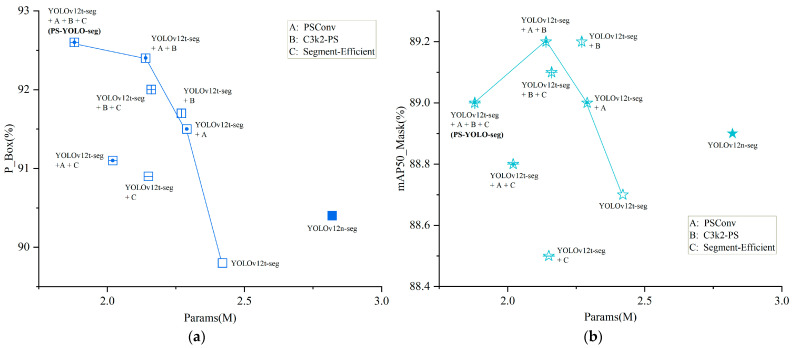
Performance curves of transition models. (**a**) $P_{box}$-Params curve; (**b**) ${mAP50}_{mask}$-Params curve.

The results of these experiments are summarized in Table 3, and the performance variation curves of the transitional models based on different combinations of improvement modules are shown in Figure 10. The generally high accuracy metrics are primarily attributed to the standardized microscopic imaging protocol and the strict area filtering preprocessing method. These techniques effectively reduced background interference and minimized false-negatives. However, this trend in the results does not affect the further comparative analysis based on these performance metrics.

In a detailed analysis, the YOLOv12t-seg model first achieves global channel scaling by adjusting the network width factor, significantly reducing the model complexity. Compared to the baseline model YOLOv12n-seg, FLOPs, the number of parameters, and model size were reduced by 5%, 14%, and 13%, respectively, while FPS increased by 11%, demonstrating the effectiveness of channel compression in lightweight design. However, this also led to a slight decrease in accuracy. Next, the independently implemented improvement modules, PSConv and C3k2-PS, both improved detection and segmentation accuracy. PSConv, with its pinwheel-shaped convolution, efficiently performed downsampling operations, while C3k2-PS better captured the textural details of microscopic mineral blocks and reduced the number of parameters slightly, while maintaining accuracy. Subsequently, the optimized segmentation head, Segment-Efficient, was integrated into the transition models (A+C and B+C), further improving the lightweight metrics while preserving the accuracy gains from PSConv. Finally, the fully integrated PS-YOLO-seg model, which combines channel scaling, downsampling operations, feature extraction modules, and efficient segmentation heads, outperformed YOLOv12n-seg in all four accuracy metrics. In addition, it achieves reductions of 20% in FLOPs, 33% in the number of parameters, and 32% in model size, along with a slight increase in FPS.

These results validate that the proposed modules effectively balance accuracy and lightweight design, enhancing both the performance and deployment feasibility of the model.

### 5.3. Comparison Experiments

**Figure 11:**
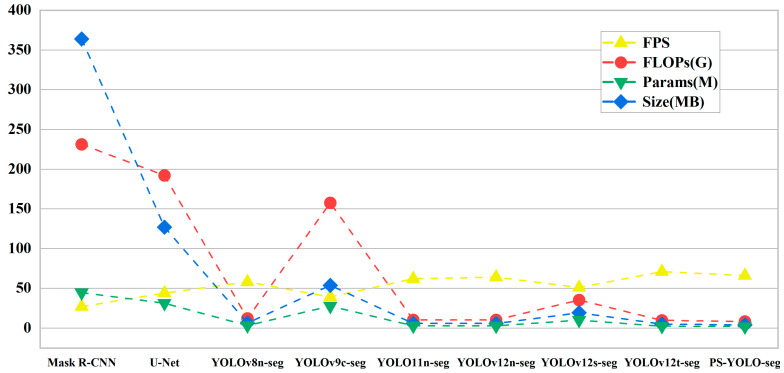
Comparison of lightweight metrics across different models.

To rigorously validate the superiority of PS-YOLO-seg, a comprehensive comparative analysis was conducted against other segmentation models. To ensure fairness, all models were trained from scratch without pre-trained weights, and their default hyperparameter configurations were adopted uniformly, without any additional optimization or adjustment. Detailed comparison results are presented in Table 4, and Figure 11 provides a comparison of the lightweight performance metrics across different models.

On the one hand, mainstream segmentation frameworks such as Mask R-CNN and U-Net (both use ResNet-50 [49] as the backbone network or encoder) achieved a strong performance, with mean segmentation accuracies of 91.3% and 91.5%, respectively, slightly outperforming our model. However, their substantial computational overhead—hundreds of GFLOPs and tens of millions of parameters—resulted in large model sizes (hundreds of megabytes) and lower FPS, making them unsuitable for deployment on edge devices. On the other hand, compared to other YOLO-based segmentation networks, the proposed model achieves a better trade-off between speed and accuracy. Specifically, when compared to commonly used models such as YOLOv8n-seg, YOLOv9c-seg, and YOLOv11n-seg, the proposed PS-YOLO-seg maintains a box precision gap within 1.5% and a mean segmentation accuracy difference within 1.6%, while achieving a reduction of 33–91% in parameter count, a 31–92% decrease in model size, and a 3–69% improvement in FPS. These results highlight the model’s balanced performance in terms of accuracy and lightweight design.

Notably, compared to YOLOv12s-seg, which is constructed with a more complex network architecture, the proposed PS-YOLO-seg exhibits a superior performance in the task of lithium ore microscopic image segmentation. This is primarily attributed to the enhanced compatibility of the PSConv and C3k2-PS modules. Specifically, PS-YOLO-seg achieves a 0.8% improvement in detection precision and a 1.8% gain in segmentation accuracy. Furthermore, by incorporating an efficient Segment-Efficient segmentation head, it reduces FLOPs by 76% and parameters by 80%, with only a 0.8% compromise in mean segmentation accuracy, clearly demonstrating its remarkable advantages in lightweight design.

A comprehensive analysis of the above results indicates that the proposed improvements not only maintain a comparable segmentation performance, but also significantly reduce model parameters and enhance computational efficiency. This makes PS-YOLO-seg particularly well-suited for efficient lepidolite microscopic image segmentation tasks, demonstrating greater potential for practical deployment.

### 5.4. Visualization Experiments

To visually demonstrate the enhanced model’s segmentation performance, four representative images from the test set were selected for comparison. Different types of mineral blocks are marked using distinct colors for detection boxes and segmentation masks, as follows: feldspar in blue, quartz in cyan, and lepidolite in gray. Key comparison areas are highlighted with red ellipses. The confidence scores (ranging from 0 to 1) in the results quantify the reliability of the detections, representing the model’s estimated probability of correctly identifying the mineral composition within the specified region.

**Figure 12:**
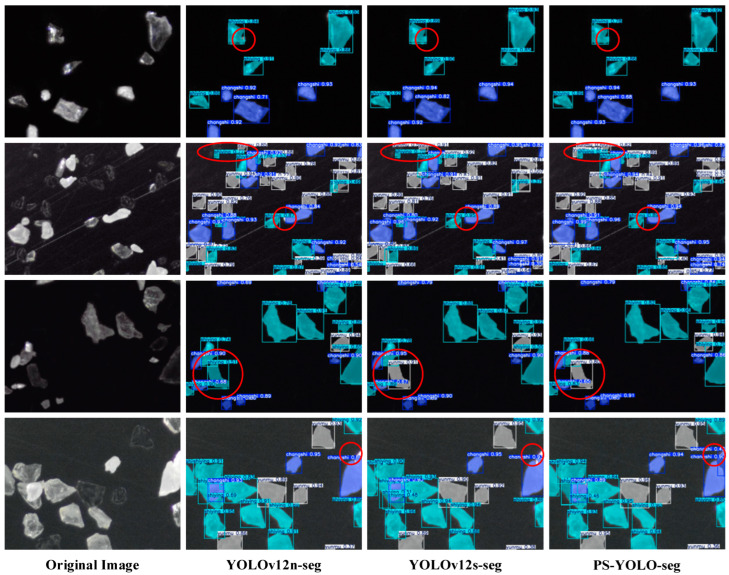
Visualization comparison of detection results. Notable areas are marked with red circles.

Figure 12 presents the segmentation results of PS-YOLO-seg alongside other baseline models. A detailed analysis is as follows: in the first row of microscopic images, the mineral blocks are relatively dispersed, and all three models correctly identify the corresponding mineral types. Notably, in the regions marked with red circles, the segmentation mask generated by YOLOv12n-seg fails to fully cover the quartz mineral block, while the other two models successfully perform complete segmentation. In terms of confidence, YOLOv12s-seg performs the best due to its deeper network structure. In the second row, the mineral blocks are more densely packed. The elliptical region at the top of the original image contains three pieces of lepidolite and one piece of quartz. All three models accurately identify the prominent quartz block. For the dimmer lepidolite, YOLOv12n-seg only identifies the larger piece, YOLOv12s-seg identifies two pieces, while PS-YOLO-seg successfully detects all of the dimmer mica pieces. Additionally, YOLOv12n-seg’s detection box for feldspar in the center of the image is too small and does not fully fit the mineral block, while the detection boxes of the other two models are more accurate. In the third row, the red circle highlights a piece of mica. However, YOLOv12n-seg mistakenly identifies it as quartz with a low confidence score, while the other two models correctly identify and segment the mica piece. YOLOv12s-seg exhibits the least confusion, and PS-YOLO-seg maintains a relatively high confidence score of 0.8. In the final row, there is some mineral block stacking. The red circle on the right edge marks two stacked feldspar blocks. The two baseline models, YOLOv12n-seg and YOLOv12s-seg, fail to detect the stacked structure, while PS-YOLO-seg successfully identifies and segments both feldspar blocks.

In summary, the test results demonstrate that, thanks to the improved modules PSConv and C3k2-PS, PS-YOLO-seg significantly enhances its ability to capture dim, small targets in microscopic environments. The model effectively addresses issues such as segmentation mask and detection box alignment, dim target recognition, and mineral block stacking. Additionally, through global channel reduction and the lightweight design of the segmentation head, PS-YOLO-seg achieves optimizations in model complexity and computational efficiency. Overall, it outperforms the original baseline model YOLOv12n-seg, demonstrating greater competitiveness and practical utility in real-world applications of lithium mineral quality analysis.

### 5.5. Generalization Experiments

**Figure 13:**
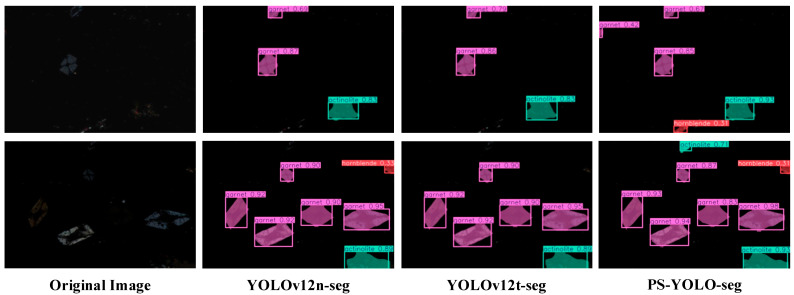
Visualization comparison on other datasets.

To further evaluate the generalization capability of the proposed model for other microscopic mineral identification tasks, the improved YOLOv12t-seg and PS-YOLO-seg models were tested on a public petrographic mineral microscopy dataset [50], comprising 1092 images, with 1116 instances of actinolite, 3880 of garnet, and 1248 of hornblende. Comparative analysis was conducted against baseline models. As shown in Table 5, PS-YOLO-seg achieves a comparable segmentation performance to YOLOv12n-seg, while exhibiting a more lightweight design. Specifically, the segmentation and detection accuracy is slightly improved compared to the baseline model, while the FLOPs are reduced by 19.6% (from 10.2G to 8.2G), the number of parameters is reduced by 33.1% (from 2.81M to 1.88M), and the model size is reduced by 32.1% (from 5.79MB to 3.93MB). A slight increase in FPS was also observed. The visualizations in Figure 13 further confirm that PS-YOLO-seg preserves competitive segmentation accuracy while retaining superior lightweight characteristics, even under low-illumination, single-polarized microscopic conditions, highlighting its strong potential for cross-domain mineral detection and practical deployment on edge devices.

## 6. Conclusions

Traditional lithium mineral analysis methods often rely on complex instrumentation and manual operations, resulting in destructive sampling, lengthy detection cycles, and high operational costs. To address these challenges and meet the dual demands of real-time processing and edge deployment in industrial settings, this study proposes a lightweight instance segmentation framework—PS-YOLO-seg—based on YOLOv12-seg, tailored for visible light microscopic lithium mineral analysis. The model incorporates several key structural improvements, as follows: global channel compression through width scaling, a novel pinwheel-shaped convolution module (PSConv) to enhance downsampling, and a feature extraction module (C3k2-PS) designed to better capture the fine-grained and low-contrast textures of mineral particles under microscopy. In addition, an efficient and streamlined segmentation head (Segment-Efficient) is introduced to further reduce model complexity. Multiple ablation studies, quantitative evaluations, and visualization comparisons confirm the effectiveness of these improvements in balancing accuracy and efficiency.

From the experimental findings, the integration of PSConv and C3k2-PS notably enhanced the model’s ability to detect subtle mineral textures, particularly under low-light and weak-contrast conditions. Meanwhile, the use of channel compression and the Segment-Efficient head significantly reduced model complexity—achieving 20% reduction in FLOPs, 33% in parameter count, and 32% in model size, along with a slight increase in FPS—while maintaining comparable accuracy to the baseline. These results demonstrate strong potential for real-world deployment on edge devices, and validate the effectiveness of the proposed design strategies.

Despite these promising results, several limitations remain and warrant further reflection. The current experiments were conducted on a large-scale, preprocessed, and standardized microscopic image dataset, which likely improved overall recognition performance and minimized accuracy differences among models. However, this also reduces visibility into how well each module adapts to more complex or uncertain environments. Moreover, the model has thus far only been tested under optical microscopy; thus, broader validation under more diverse imaging conditions (e.g., polarized light or multispectral imaging) is needed. In addition, the training parameters were selected based on common practice rather than systematic optimization, which will be a focus in future work.

Moving forward, we plan to refine this approach by expanding it to cover more complex mineral mixtures, including lepidolite and other coexisting ores, and explore multimodal fusion techniques to improve robustness. Ultimately, our goal is to enable rapid identification and a purity assessment of industrial-grade lithium minerals using deep learning, thereby facilitating precise resource development, reducing labor costs, and advancing the automation and intelligence of mineral detection and classification.

## Figures and Tables

**Table 1:** Comparison of scaling factors in YOLOv12-seg models.

Network	Widen Factor	Deepen Factor	Max Channels	Params (M)
YOLOv12n-seg	0.25	0.5	1024	2.82
YOLOv12s-seg	0.5	0.5	1024	9.89
YOLOv12t-seg	0.125	0.5	512	2.42

**Table 2:** Details of dataset partitioning.

	Training Set	Validation Set	Test Set	Total
Total	11,159	3025	1498	15,682
Feldspar	3407	932	487	4826
Quartz	4336	1155	589	6080
Lepidolite	3416	938	422	4776

Note: The sum of each row represents the total number of instances in that category, while each column represents the total number of instances within that set.

**Table 3:** Results of ablation experiments.

Model	A	B	C	P_box_ (%)	P_mask_ (%)	mAP50_box_ (%)	mAP50_mask_ (%)	FLOPs (G)	FPS	Params (M)	Size (MB)
YOLOv12n-seg				90.4	91.1	94.2	88.9	10.2	64	2.82	5.79
YOLOv12t-seg				89.8	90.1	93.7	88.7	9.7	71	2.42	4.98
Transition Model	√			91.5	91.4	94.3	89.0	9.6	60	2.29	4.77
		√		91.7	91.8	94.6	89.2	9.4	62	2.27	4.68
			√	90.9	90.8	93.9	88.5	8.5	69	2.15	4.43
	√	√		92.4	92.2	94.8	89.2	9.4	61	2.14	4.48
	√		√	91.1	91.2	94.1	88.8	8.5	64	2.02	4.23
		√	√	92.0	91.5	94.3	89.1	8.7	68	2.16	4.13
PS-YOLO-seg	√	√	√	92.6	91.6	94.4	89.0	8.2	66	1.88	3.93

**Table 4:** Comparison results with other segment models.

Model	P_box_ (%)	P_mask_ (%)	mAP50_box_ (%)	mAP50_mask_ (%)	FLOPs (G)	FPS	Params (M)	Size (MB)
Mask R-CNN [44]	93.9	92.5	95.4	91.3	231.2	27	44.5	364
U-Net [45]	93.3	92.8	96.1	91.5	192.0	44	31.1	127
YOLOv8n-seg [46]	92.5	93.0	95.8	89.9	12.1	58	3.26	6.48
YOLOv9c-seg [47]	94.1	93.5	96.0	90.9	157.6	39	27.6	53.6
YOLO11n-seg [48]	92.8	92.7	95.5	90.3	10.3	62	2.84	5.74
YOLOv12n-seg	90.4	91.1	94.2	88.9	10.2	64	2.82	5.79
YOLOv12s-seg	91.8	89.8	95.2	89.8	35.2	51	9.89	19.3
YOLOv12t-seg	89.8	90.1	93.7	88.7	9.7	71	2.42	4.98
PS-YOLO-seg	92.6	91.6	94.4	89.0	8.2	66	1.88	3.93

**Table 5:** Comparison results on other datasets.

Model	P_box_ (%)	P_mask_ (%)	mAP50_box_ (%)	mAP50_mask_ (%)	FLOPs (G)	FPS	Params (M)	Size (MB)
YOLOv12n-seg	83.2	70.9	88.1	63.1	10.2	80	2.81	5.79
YOLOv12t-seg	82.3	68.7	86.8	62.8	9.7	87	2.42	4.96
PS-YOLO-seg	82.5	71.1	88.4	63.3	8.2	83	1.88	3.93

## Data Availability

The data presented in this study are available on request from the corresponding author.

## Abbreviations

The following abbreviations are used in this manuscript:

XGBoost	Extreme Gradient Boosting
CV	Computer Vision
A2C2f	Area Attention and Cross-Stage Partial Structure with 2 Convolutions
ELAN	Efficient Layer Aggregation Networks
MLP	Multilayer Perceptron
C3k2	Cross-Stage Partial Structure with Convolution 3 and kernel size 2
C3	CSPDarknet53 with 3 Convolutions
C2f	Cross-Stage Partial Structure with 2 Convolutions
